# Effects of a calorie-restricted MIND diet and probiotic supplementation on fertility, sexual function, and quality of sexual life in overweight and obese men: protocol for a double-blind randomized clinical trial

**DOI:** 10.1186/s13063-026-09891-1

**Published:** 2026-07-13

**Authors:** Ali Shamsi-goushki, Mohammad Reza Mahmoodi, Sareh Ashourzadeh, Majid Khadem-Rezaiyan, Reza Rezvani

**Affiliations:** 1 Department of Nutrition, Faculty of Medicine, Mashhad University of Medical Sciences, Azadi Square, Campus of University, Mashhad, Iran; 2 Physiology Research Center, Institute of Neuropharmacology & Department of Nutrition, Faculty of Health, Kerman University of Medical Sciences, Kerman, Iran; 3 Department of Obstetrics and Gynecology, Faculty of Medicine, Kerman University of Medical Sciences, Kerman, Iran; 4 Department of Community Medicine, Faculty of Medicine, Mashhad University of Medical Sciences, Mashhad, Iran

**Keywords:** MIND diet, Probiotic supplementation, Fertility, Overweight, Sexual function, Quality of sexual life

## Abstract

**Background:**

MIND diets and probiotics have the potential to positively affect infertility in obese and overweight men, but their effects are not yet fully elucidated. Therefore, this study aims, for the first time, to address this gap by evaluating the effects of a calorie-restricted MIND diet and probiotic supplementation on fertility, sexual function, and quality of sexual life in overweight and obese men.

**Methods:**

This study will be conducted as a double-blind randomized clinical trial on 84 overweight and obese men in Kerman for 3 months. Participants will be randomly allocated to one of the following four groups: (1) the group receiving the MIND diet with restricted calories + probiotic supplement; (2) the group receiving the MIND diet with restricted calories + placebo; (3) the group receiving a standard low-calorie diet + probiotic supplement; (4) the group receiving a standard low-calorie diet + placebo. Before the intervention and after 3 months, the study variables will be compared between groups. Anthropometric evaluation, spermogram evaluation, serum levels of androgens, sexual function, and quality of sexual life will be assessed at the beginning and at the end of the study.

**Discussion:**

This trial will provide valuable evidence regarding the potential synergistic effects of the MIND diet and probiotic supplementation in managing male infertility associated with obesity. By evaluating both clinical fertility markers and patient-reported outcomes such as sexual function and quality of life, the study findings are expected to contribute to a better understanding of the role of lifestyle interventions in reproductive health, potentially offering a sustainable therapeutic approach for this population.

**Ethical aspects:**

All the work procedures will be carried out in accordance with the guidelines recommended by the Ethics Committee of Mashhad University of Medical Sciences (IR.MUMS.REC.1402.341).

**Trial registration:**

Iranian Registry of Clinical Trials IRCT20240313061277N1. Registered on 31 March 2024.

**Supplementary Information:**

The online version contains supplementary material available at https://doi.org/10.1186/s13063-026-09891-1.

## Introduction

Infertility is defined as the inability to conceive after 12 months of unprotected sex, or 6 months for women over the age of 35 [1]. Male infertility refers to a man’s inability to impregnate a fertile woman after at least 1 year of unprotected intercourse [2]. Globally, infertility affects approximately 48.5 million couples [3, 4]. The quality of semen plays a crucial role in fertility and the development of normal embryos [5]. Evidence suggests that human semen quality has declined by 50–60% over the past four decades [6]. Studies show that globally, 13% of women and 10% of men experience infertility. In the USA, the prevalence of infertility is 15.5%, while in Iran, it is 7.88%, with 50% of cases attributed to male factors [7]. The treatment of infertility in men is more challenging than in women, particularly in developing countries where the high cost of treatment poses significant barriers [8]. Various factors, including genetics, endocrine disorders, obesity, unhealthy diets, and demographic factors such as age, smoking, and excessive alcohol consumption, are known to negatively affect sperm quality and contribute to male infertility [9, 10]. Among these factors, nutrition represents a modifiable determinant with significant preventive and therapeutic potential [6, 11].

Nutrition can negatively or positively affect sperm quality, and this effect depends on the quantitative and qualitative aspects of a diet, such as the caloric content of each macronutrient, micronutrient, and antioxidant in that diet [10]. Following the Western food pattern, excessive consumption of simple carbohydrates, saturated and trans fatty acids, and a reduction in fiber intake has a negative effect on sperm quality and, consequently, on the fertilization process [12]. On the other hand, healthy dietary patterns, such as the Mediterranean diet, are clearly associated with better sperm quality, suggesting that nutritional interventions can play a key role in maintaining male fertility [10, 13]. The Mediterranean diet is characterized by low consumption of red meat, the use of olive oil instead of animal fats as fat sources, the consumption of seafood and poultry, moderate amounts of red wine, and significant amounts of fresh fruits and vegetables. The presence of polyphenols, antioxidants, omega-3 fatty acids, B-complex vitamins, and zinc in this diet has attracted the attention of the scientific community due to their antioxidant and anti-inflammatory properties [14]. Indeed, several phytochemical compounds present in the Mediterranean diet can act through various mechanisms, including the inhibition of reactive oxygen species (ROS), chelation of toxic metals, anti-inflammatory effects, epigenetic enhancement of detoxification, reduction of the harmful effects of environmental pollutants, and positive effects on genes or enzymes. These mechanisms can have a positive effect on sperm quality parameters [14]. The MIND diet, which combines elements of the Mediterranean and DASH dietary patterns, may therefore provide similar reproductive benefits in addition to its established neuroprotective effects.

The Western diet has played a crucial role in increasing obesity rates, which has had a significant impact on fertility by changing hormone levels, sperm function, and gamete molecular composition. However, the molecular mechanisms responsible for the causal links between obesity and male infertility are not fully elucidated. Obesity has been shown to disrupt various components of the hypothalamic-pituitary–gonadal axis, which is mainly related to decreased testosterone levels and decreased sperm count [10, 15].

Probiotics are one of the compounds that can have a positive effect on sperm quality [16, 17]. Probiotics are defined as “live microorganisms” that, when administered in sufficient amounts, are beneficial to the health of the host [18]. The use of probiotics is increasing worldwide as a treatment for many different diseases and disorders. In the field of reproduction, the role of the microbiome in women’s health has been widely studied. Several mechanisms have been suggested for the way probiotics work, such as the elimination of pathogens and the production of inhibitors such as bacteriocins and organic acids can be mentioned [19, 20]. The effects of probiotics as powerful antioxidants have also been demonstrated in several studies [21, 22]. These properties suggest that probiotic supplementation may complement dietary interventions aimed at improving reproductive outcomes.

Since the prevalence of infertility in men is high and studies on the combined effect of the MIND diet with limited calories and probiotic supplementation on male infertility are scarce, the purpose of this novel study is to determine the impact of the MIND diet with limited calories and probiotic supplementation on spermogram parameters, serum levels of androgens, sexual function, and quality of sexual life in overweight and obese men. The MIND diet was selected as an intervention because of its anti-inflammatory and antioxidant properties, which are hypothesized to improve sperm quality, androgen levels, and overall reproductive health in overweight and obese men. A calorie-restricted standard diet was chosen as the comparator to distinguish the specific effects of the MIND diet from those of general calorie restriction.

## Objectives

### Primary outcome

To determine the effect of the MIND diet with restricted calories and probiotic supplementation on spermogram parameters, serum androgen levels, sperm DNA fragmentation, sexual performance, and quality of sexual life in overweight and obese men.

### Secondary outcomes

- To determine the effect of the MIND diet with restricted calories and probiotic supplementation on spermogram parameters in overweight and obese men.
- To evaluate the impact of the MIND diet with restricted calories and probiotic supplementation on serum androgen levels in overweight and obese men.
- To assess the effect of the MIND diet with restricted calories and probiotic supplementation on the sperm DNA fragmentation index in overweight and obese men.
- To examine the influence of the MIND diet with restricted calories and probiotic supplementation on sexual performance in overweight and obese men.
- To evaluate the effect of the MIND diet with restricted calories and probiotic supplementation on the quality of sexual life in overweight and obese men.

## Trial design

This study is a double-blind, randomized, placebo-controlled, parallel-group clinical trial with an allocation ratio of 1:1:1:1. The framework of the trial is based on a superiority design. It aims to evaluate whether a calorie-restricted MIND diet and probiotic supplementation, alone or in combination, produce superior effects on fertility, sexual function, and quality of sexual life compared with a standard calorie-restricted diet and/or placebo in overweight and obese men.

## Methods and analysis

### Study setting, oversight, and monitoring

**Fig. 1:**
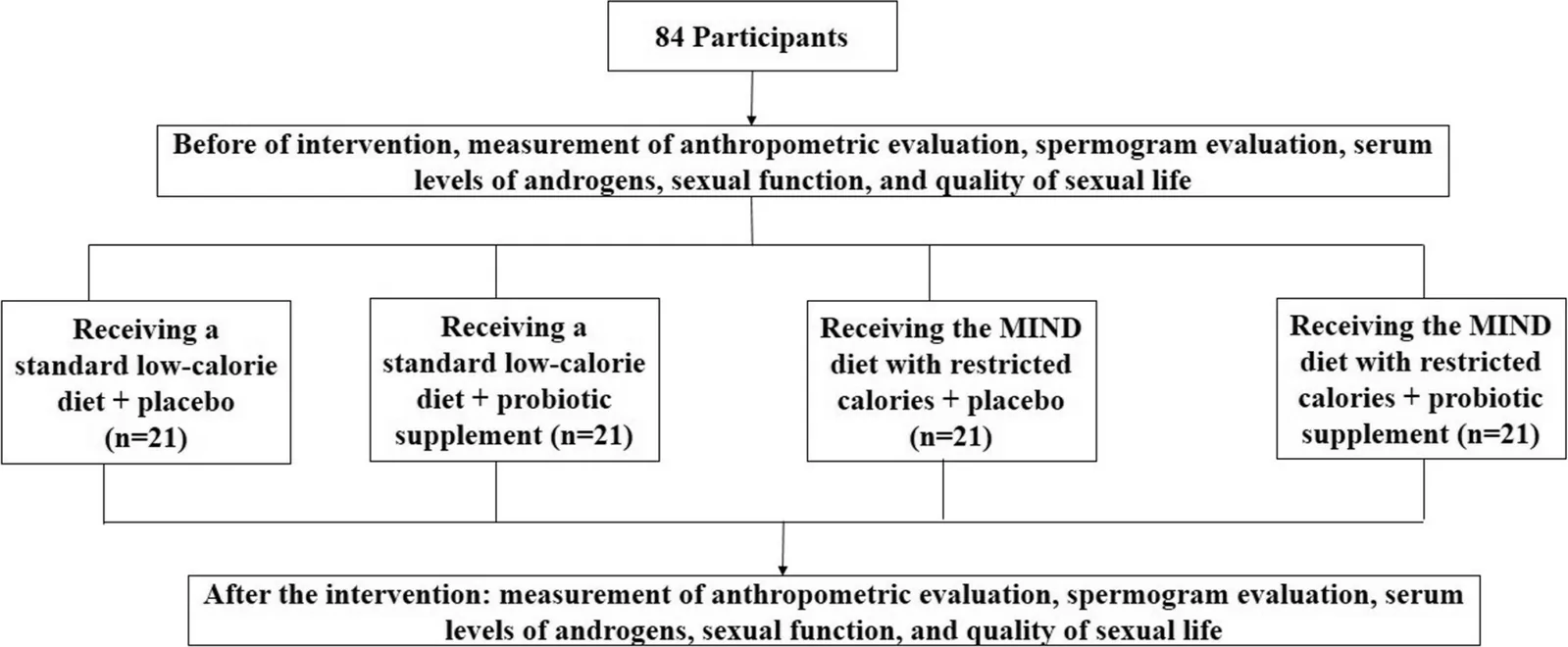
Study flowchart

The present study is a randomized clinical trial that will be conducted over 3 months on 84 overweight and obese men referred to the KERCADRS cohort center in Kerman and the Afzalipur Infertility Treatment Research Center in Kerman. Participation in the study will be completely voluntary. A written consent form will be obtained from the 84 participants who volunteer, meet the inclusion criteria, and do not meet the exclusion criteria after a verbal explanation of the study’s methods, goals, advantages, and limitations. Before starting the study, participants will complete a general information questionnaire that includes demographic information, occupation, education, physical activity, use of special medications, medical conditions, and a 24-h dietary recall form to determine their eating patterns and habits. Additionally, the anthropometric indices of all participants will be measured. Blood and semen samples will be collected from all participants to evaluate morphological indicators, sperm motility, percentage of live sperm, sperm DNA fragmentation index, and androgen hormones (total testosterone, free testosterone, and dehydroepiandrosterone). The timeline of the trial and study flow chart of enrollment, allocation, intervention, and assessment are presented in Table 1 and Fig. 1, respectively.

**Table 1:** Timeline of the trial

Explanation of the trial activities	Time (months)															
	1	2	3	4	5	6	7	8	9	10	11	12	13	14	15	16
Material preparation	*	*	*	*	*											
Recruitment						*	*	*	*							
Clinical assessments at baseline						*	*	*	*							
Nutritional assessments at baseline						*	*	*	*							
Semen evaluation and biochemical assessments at baseline						*	*	*	*							
Anthropometric assessment at baseline						*	*	*	*							
Evaluation of sexual function and quality of sexual life at baseline						*	*	*	*							
Intervention									*	*	*	*	*	*		
Clinical assessments after intervention									*	*	*	*	*	*		
Nutritional assessments after intervention									*	*	*	*	*	*		
Semen evaluation and biochemical assessments after intervention									*	*	*	*	*	*		
Anthropometric assessment after intervention									*	*	*	*	*	*		
Evaluation of sexual function and quality of sexual life after intervention									*	*	*	*	*	*		
Data analysis														*	*	
Writing the final report of the trial																*
The expected time	*	*	*	*	*	*	*	*	*	*	*	*	*	*	*	*

### Eligibility criteria

#### Inclusion criteria

The criteria for entering the study will be included: willingness to participate in the study, being a married man, age between 20 and 45 years, overweight or obese men with a body mass index (BMI) greater than 25, and no use of probiotic supplements in the last 3 months.

#### Non-entry criteria

Non-entry criteria will be included having azoospermia, varicocele, cryptorchidism, or microorchidism; a history of vasectomy; use of medications that harm spermatogenesis, such as sulfasalazine; tobacco, alcohol, or drug use; infections or anatomical abnormalities of the reproductive system; and having cancer or chronic diseases.

#### Exclusion criteria

Exclusion criteria will include the following: unwillingness to continue the study at any stage of the project, following special diets, failure to adhere to the prescribed diet, or not taking the probiotic supplements.

### Consents

Informed consent will be obtained by the research team and has received training in ethical principles and informed consent procedures. All personal information of potential and enrolled participants will be collected using coded identifiers. All records will be maintained in accordance with institutional and national data protection regulations.

### Randomization

Randomization will be performed using computer-generated block randomization (Sealedenvelope.com) by an independent statistical consultant. Allocation concealment will be ensured using sequentially numbered, opaque, sealed envelopes (SNOSE). Block sizes will not be disclosed to investigators involved in enrollment.

### Blinding

Blinding will be achieved using sequentially numbered, opaque sealed envelopes (SNOSE). The generated sequence will be encrypted, and after verifying the inclusion and exclusion criteria and obtaining informed consent, the researcher will take an envelope from the sequence, write the participant’s name on the back, and then open it. The participant will be assigned to the group specified inside the envelope.

Participants in the study will be randomly assigned to one of four groups. The supplement and placebo will be provided in identical packaging to ensure that neither the participants nor the researchers are aware of the contents. Additionally, the individual responsible for evaluating the results will remain blinded to the group allocations.

### Final sample size

Using the findings of the study by Helli et al., who examined the effects of probiotics on sperm parameters, oxidative stress index, inflammatory factors, and sex hormones in infertile men [23], the live sperm index in the intervention and control groups was reported as 12.17 ± 62.43 and 8.9 ± 52.6, respectively. Taking into account an alpha error of 5% and a power of 80%, the required sample size for each group is 19 participants. It should be noted that the use of ejaculate volume, total sperm count, and motility grade a + b + ca indicators from the mentioned study resulted in a much smaller sample size of 7 participants per group. Due to the existence of 4 groups, 19 participants per group, and considering a 10% attrition rate, a final total of 21 participants will be included in each group (84 participants in total). Where values were as follows; *α* = 0.05, *β* = 0.20, µ1 = 62.43, µ2 = 52.6, SD1 = 12.17, and SD2 = 8.9.





The allocation sequence will be generated by a statistical consultant using computer-generated random numbers through Sealedenvelope.com. Block randomization will be applied to ensure equal group sizes. No stratification factors will be used. To maintain allocation concealment and reduce predictability, details of block sizes will be documented in a separate file that will not be accessible to investigators involved in participant enrollment or intervention assignment.

### Study interventions

#### The group receiving the MIND diet with restricted calories + probiotic supplement for 3 months

To adjust the diet, the energy required by the individual will be calculated based on the Mifflin formula, with 500–750 kcal deducted from it. The macronutrient distribution will include 50–55% carbohydrates, 30% fat, and 15–20% protein. The MIND diet emphasizes ten components that are beneficial for the brain, including green leafy vegetables, other vegetables, nuts, berries, legumes (especially beans), whole grains, fish (more than one serving per week), poultry (more than two servings per week), olive oil, and wine. Five unhealthy components for the brain, including red meat, butter and margarine, cheese, cakes and sweets, fast food, and fried foods, will be limited in this diet. In this study, the consumption of wine will not be considered due to cultural and religious restriction in Iran; instead, it is recommended to consume raisins and currants. The participants’ diet will include 3 main meals and 3 snacks.

### The group receiving the standard low-calorie diet + probiotic supplement for 3 months

To adjust the low-calorie diet, the energy required by the individual will be calculated based on the Mifflin formula, with 500–750 kcal deducted from it. The macronutrient distribution will include 50–55% carbohydrates, 30% fat, and 15–20% protein. Then, the units of each food group will be determined based on the standard diet. The participants’ diet will include 3 main meals and 3 snacks.

### MIND diet group with restricted calories + placebo

#### The group receiving the standard low-calorie diet + placebo

At the beginning of the study, all participants will be instructed on how to take the supplements and complete the questionnaires. This instruction will be delivered by a PhD student and the clinical advisor of the thesis, ensuring that participants understand the procedures and can adhere correctly to the intervention protocol. At the beginning and end of the study, 5 mL of blood samples (after 12 h of fasting) and semen samples will be collected for laboratory tests. Patients will be reminded to adhere to the lifestyle intervention program (diet and physical activity) through weekly phone calls and monthly face-to-face meetings. At the beginning and end of the intervention, participants will complete a 3-day food record and a supplement intake questionnaire to monitor adherence to the prescribed diet and supplement regimen. These procedures allow the research team to track compliance, identify any protocol deviations, and provide guidance as needed to ensure accurate implementation of the intervention.

### Data collection and management

#### Nutritional assessment

A 3-day food record (2 weekdays and 1 weekend day) will be collected from each participant at the beginning and at the end of the study. Adherence to supplementation will also be assessed by counting the returned capsules at the midpoint and at the end of the trial. The compliance rate will be calculated using the following formula, with poor adherence defined as less than 90%:

Compliance rate (%) = (capsules taken/capsules prescribed) × 100.

Protocol deviations will be documented throughout the trial by the research team during weekly phone calls, monthly follow-up visits, and capsule count assessments. Major deviations, including poor adherence to the dietary prescription or supplement intake, withdrawal from the intervention, or use of prohibited medications or supplements, will be recorded in the study files. Participants with protocol deviations will remain in the study and will be included in the final analysis according to the intention-to-treat principle. The type and frequency of protocol deviations will be reported descriptively.

### Delivery of intervention

All intervention procedures and data collection will be conducted by two qualified members of the research team: one author who is a PhD student, and another author holding a Doctorate in Reproductive Health. Both team members have been trained in study procedures, dietary counseling, supplement administration, and ethical considerations, ensuring accurate implementation of the interventions and reliable data collection.

### Data management and monitoring

The study will be overseen by a clinical trial monitor, who will periodically review the trial progress to ensure that participant rights and well-being are protected, and that the study protocol, ethical guidelines, and regulatory standards are being followed. The monitor will also confirm that all essential documents are available and that the collected data are accurate and properly recorded. One of the investigators will be responsible for checking data coding, security, and storage, and will verify data entry and data values twice to ensure accuracy. In the event that a participant reports an adverse event, additional information will be collected to determine whether exclusion from the trial is necessary. Unblinding may be permitted in such cases according to the criteria set by the Medical Ethics Committee.

### Semen evaluation

Two semen samples will be collected from the referred men, 3 months apart. Samples will be collected after 3 days of abstinence from sexual intercourse through masturbation. The prepared semen samples will be incubated at 37 °C for about half an hour to transform from a lumpy form to a flowing liquid. About 200 µL of the seminal fluid sample will be examined to check sperm parameters based on the standards of the World Health Organization [24]. Individuals with abnormal spermatogram test results will be referred to a urologist. The amount of motility will be estimated using computer analysis of semen. Additionally, in the evaluation of sperm motility based on WHO criteria, the studied samples will be divided into four groups based on the type of motility: sperm with fast progressive motility, sperm with slow progressive motility, sperm with non-progressive motility, and non-motile sperm. Further microscopic tests will be performed to evaluate and determine parameters such as sperm concentration per milliliter of semen, percentage of live sperm, sperm morphology, and sperm DNA fragmentation index.

### Outcomes

#### Biochemical evaluation

A venous blood sample of 10 mL will be taken from the participants once before the start of the study and a second time after 3 months. The resulting serum will be used to measure the following indicators: total testosterone, free testosterone, and dihydroepiandrosterone, which will be measured using the RIA method and the Monobind kit.

#### Anthropometric assessment

Anthropometric measurements will include weight, height, body mass index, waist circumference, hip circumference, neck circumference, and the ratio of waist circumference to hip circumference. Weight will be recorded by a nutrition expert with minimal clothing and without shoes using a digital scale with an accuracy of 100 g. Height will be measured with an accuracy of 1 cm using a tape measure while standing next to the wall, without shoes, with the shoulders in a neutral position, and looking straight ahead. Body mass index will be calculated by dividing weight (in kilograms) by the square of height (in meters squared). Waist circumference will be measured transversely at the smallest point, from the navel to the end of the sternum bone, while hip circumference will be measured transversely at the largest diameter of the hip region. Neck circumference will be measured just below the ridge of the larynx. The ratio of waist circumference to hip circumference will then be calculated by dividing waist circumference (cm) by hip circumference (cm).

Because the measurements will be taken in a situation where the subjects are wearing light clothing, they will be asked to remove any clothes that might affect the shape of the body and waist. Additionally, the person taking the measurements will be asked to carefully check the pressure applied by the tape measure to ensure that no pressure is being exerted on the body (the tape measure will neither be loose nor tight).

At the end of the study (after 3 months), the anthropometric indices of the participants will be measured and calculated again. Additionally, their blood and semen samples will be collected to check the levels of androgens, sperm motility, and morphology, and the results will be recorded in the form related to general, laboratory, and anthropometric information. Follow-up of the participants, to monitor capsule consumption, physical activity, and prevent sample loss, will be done by phone once a week. At the end of the study, by counting the remaining capsules, the level of compliance with capsule intake will be assessed, and by filling out the physical activity questionnaire, the level of physical activity will be evaluated.

#### Evaluation of sexual function and quality of sexual life

Sexual performance will be evaluated using the Men’s Sexual Performance Questionnaire, and the quality of sexual life will be assessed using the Men’s Quality of Sexual Life Questionnaire (SQOL-M). Sexual Performance Questionnaire (BSFI): This questionnaire contains 11 questions that examine men’s sexual performance in 5 parts: (1) Sexual desire: Questions 1 and 2; (2) Erection: Questions 3, 4, and 5; (3) Ejaculation: Questions 6 and 7; (4) Problem assessment: Questions 8, 9, and 10; and (5) Overall satisfaction: Question 11.

Each question will have five options to choose from, and each option will be assigned a score of 0–4. Then, the average score for each of the 5 parts will be calculated, and the score of each part will be equal to the total score of its subsets. In fact, a lower score will indicate lower sexual performance. If any question is left unanswered, the average score of that question will be used. The Men’s Sexual Performance Survey tool was first developed in 1995 by O’Leary as a self-report scale to measure current sexual performance [25]. The content validity of this questionnaire has been confirmed in the study by Alizadeh Cherandabi et al. [26], and its reliability has been calculated using Cronbach’s alpha between 0.81 and 0.92. The reliability of this tool in the research population of this study will be determined by measuring Cronbach’s alpha and ICC (intraclass correlation) through retesting 20 participants after 2 weeks.

Men’s Sexual Quality of Life Questionnaire (SQOL-M): The Men’s Sexual Quality of Life Questionnaire specifically examines the quality of men’s sexual life. This self-report questionnaire aims to measure the quality of men’s sexual life, focusing on the dimensions of sexual self-confidence, emotional health, and interpersonal communication during the past 4 weeks [27]. The Persian version of the Men’s Quality of Life Questionnaire contains 11 items, similar to the original version. The items use a 6-point Likert scale, ranging from completely agree (score 1) to completely disagree (score 6), with a score range of 11–66, where a higher score indicates a better quality of life. The reliability of the tool was obtained using the internal consistency method, with Cronbach’s alpha for the entire tool being 0.94. The intraclass correlation (ICC) obtained from the retest on 20 qualified men with an interval of 2 weeks was 0.95, which indicates the instrument’s stability [28].

### Statistical analysis

In this study, the obtained data will be analyzed using SPSS 18 software. Quantitative normal variables will be displayed as mean ± standard deviation, non-normal quantitative variables will be displayed as median ± interquartile range, and qualitative variables will be displayed as frequency percentages. In order to increase the accuracy of the results, all analyses will be performed according to the intention-to-treat (ITT) principle. This approach includes all participants in the groups to which they were originally assigned, regardless of adherence or protocol deviations. For missing outcome data, the pattern and extent of missingness will first be assessed. If data are considered to be missing at random, multiple imputation will be used for the primary analysis. A sensitivity analysis using complete-case data will also be performed to evaluate the robustness of the findings. The distribution of qualitative variables among the groups will be done using the Chi-Square test. For intergroup and intragroup comparisons of quantitative variables with normal distribution, the two independent sample *T* test and paired *T* test will be used, respectively. If the distribution of the variables is not normal, the Mann–Whitney *U* and Wilcoxon rank tests will be used, respectively. In order to reduce the effect of confounders, at the beginning or during the study, covariance analysis or regression models will be used.

### Research ethics approval

The trial conduct and data collection procedures will be audited at least once by the Ethics Committee of Mashhad University of Medical Sciences. The auditing process will be independent from the investigators and the sponsor, focusing on informed consent, adherence to the protocol, and accuracy of data management. The study protocol, informed consent forms, and all related documents will be submitted for review and approval to the Ethics Committee of Mashhad University of Medical Sciences before the start of participant recruitment. Any subsequent amendments to the protocol will also be reviewed and approved by the Ethics Committee prior to implementation.

## Discussion

The global burden of illness linked to overweight and obesity has been steadily increasing. According to the WHO, the number of overweight and obese individuals nearly tripled between 1975 and 2016 [29]. Obesity negatively impacts both conventional and biofunctional sperm parameters and induces epigenetic alterations that can be transmitted to offspring. Additionally, obesity is associated with dysregulation of adipocyte function and microenvironmental inflammatory processes, contributing to related disorders. Dysregulated adipokines disrupt insulin signaling, which may further impair testicular function [30]. Obesity and its underlying mediators negatively affect semen parameters, including sperm concentration, motility, viability, and proper morphology. Furthermore, obesity promotes apoptosis, impairs chromatin condensation, induces DNA fragmentation, and causes epigenetic modifications that can be transmitted to offspring [31]. Dietary interventions and supplements may help reduce obesity-related reproductive impairments and enhance male fertility. The Mediterranean-Dietary Approaches to Stop Hypertension (DASH) Intervention for Neurodegenerative Delay (MIND) diet effectively promotes brain health while discouraging unhealthy food choices.

The MIND diet includes nutrient-rich foods such as leafy green vegetables, berries, nuts, whole grains, fish, chicken, and olive oil, which provide essential nutrients like vitamin E, folate, flavonoids, and carotenoids that support brain health. It also limits the intake of foods associated with cognitive decline, including red meat, butter, cheese, pastries, and fried foods [32]. Numerous studies have demonstrated that the Mediterranean, DASH, and MIND diets positively influence mental health outcomes, such as reducing anxiety and depression [33, 34]. The anti-inflammatory and antioxidant properties of the MIND diet may also benefit hormonal and metabolic functions relevant to reproduction [35]. There is evidence showing that probiotics can decrease inflammation and oxidative stress, increase androgen production, and improve testicular spermatogenic function by restoring intestinal microbiota equilibrium in animal models of obesity [36]. Thus, combining the MIND diet with probiotic supplementation may provide additive benefits for fertility in obese men. We anticipate that the intervention will improve sperm quality, reduce DNA fragmentation, and enhance chromatin integrity. Furthermore, we hypothesize that androgen levels (total and free testosterone and DHEA) will increase due to the anti-inflammatory and antioxidant effects of the intervention. Probiotics are also expected to enhance testicular function by reducing oxidative stress and systemic inflammation, which are commonly associated with obesity. The calorie-restricted diet is expected to promote weight loss, improve waist-to-hip ratios, and enhance overall anthropometric outcomes.

Based on the BSFI questionnaire, sexual performance is likely to improve in domains including desire, erection, ejaculation, and overall satisfaction. Similarly, the SQOL-M questionnaire is expected to reflect enhancements in the quality of sexual life, such as greater self-assurance, improved emotional well-being, and better interpersonal communication.

Furthermore, the study may provide insights into the mechanisms by which probiotics and the MIND diet modulate gut microbiota, reduce obesity-linked epigenetic changes, and improve metabolic health. Given the exploratory nature of this trial, the findings are intended to inform future non-pharmacological strategies for managing male infertility associated with obesity.

## Strengths and limitations

As this manuscript presents a clinical trial protocol, the future findings should be interpreted considering the exploratory nature of the study. Potential limitations include the relatively short duration of the intervention, possible variability in participants’ adherence to the dietary recommendations and supplement intake, and the reliance on self-reported dietary records, which may be subject to reporting bias. Nevertheless, the randomized, double-blind, placebo-controlled design, structured follow-up procedures, and predefined adherence monitoring strategies are expected to enhance the methodological rigor and internal validity of the trial.

## Trial status

On March 31, 2024, this trial was registered on ClinicalTrials.gov with the ID IRCT20240313061277N1. Enrollment for the trial began 6 months ago. Data collection is expected to take approximately 16 months.

## Supplementary Information

Additional file 1: SPIRIT checklist.
